# A primer to directed evolution: current methodologies and future directions

**DOI:** 10.1039/d2cb00231k

**Published:** 2023-01-27

**Authors:** Lara Sellés Vidal, Mark Isalan, John T. Heap, Rodrigo Ledesma-Amaro

**Affiliations:** a Imperial College Centre for Synthetic Biology, Imperial College London London SW7 2AZ UK r.ledesma-amaro@imperial.ac.uk; b Department of Bioengineering, Imperial College London London SW7 2AZ UK; c Department of Life Sciences, Imperial College London London SW7 2AZ UK; d School of Life Sciences, The University of Nottingham, University Park Nottingham NG7 2RD UK

## Abstract

Directed evolution is one of the most powerful tools for protein engineering and functions by harnessing natural evolution, but on a shorter timescale. It enables the rapid selection of variants of biomolecules with properties that make them more suitable for specific applications. Since the first *in vitro* evolution experiments performed by Sol Spiegelman in 1967, a wide range of techniques have been developed to tackle the main two steps of directed evolution: genetic diversification (library generation), and isolation of the variants of interest. This review covers the main modern methodologies, discussing the advantages and drawbacks of each, and hence the considerations for designing directed evolution experiments. Furthermore, the most recent developments are discussed, showing how advances in the handling of ever larger library sizes are enabling new research questions to be tackled.

## Abstract 2

This review summarises the methods available for directed evolution, including mutagenesis and variant selection techniques. The advantages and disadvantages of each technique are presented, and future challenges in the field are discussed.

## Graphical abstract

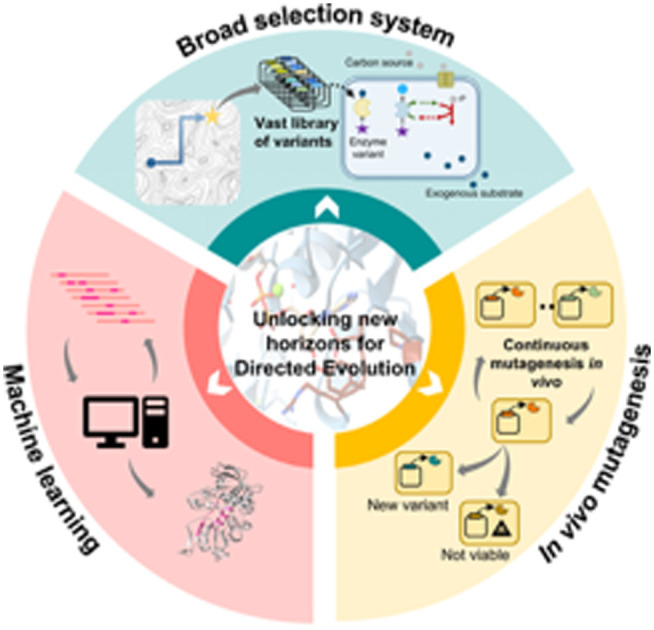

## Introduction

Enzymes have attracted increasing interest from industry as more efficient and less costly alternatives to other synthetic chemistry tools, such as transition metal catalysts or organocatalysts.^1^ However, while the repertoire in nature provides a vast variety of biocatalysts, it is frequently the case that natural enzymes need to be tailored by protein engineering in order to maximise their performance for specific applications. The same applies to biomolecules performing other functions, such as binding partners (including antibodies), fluorescent or bioluminescent macromolecules and biocatalysts acting on other macromolecules.

Two main approaches can be taken to carry out protein engineering: rational design and directed evolution. Rational design involves performing chosen point mutations, insertions or deletions in the coding sequence, and mutation choice is typically based on structural and functional information about the target biomolecule. Nonetheless, the sequence–structure–function relationship is often difficult to predict accurately, particularly at the single residue level. Additionally, reliable structural information is frequently not available for the protein of interest and, while progress is being made in methods for protein structure prediction thanks to artificial intelligence,^2^ these still remain rather limited, especially for larger proteins and macromolecular complexes.^3^ Often therefore, rationally designed mutations do not have the desired effect.

Directed evolution, on the other hand, bypasses the need to determine specific mutations *a priori* by mimicking the process of natural evolution in the laboratory.^4,5^ In nature, mutations which are beneficial for individuals are iteratively selected through numerous generations. In directed evolution, this process takes place on a much shorter timescale, and generates biomolecules that suit human-defined applications.

The first *in vitro* evolution experiments can be traced back to the 1960s. In a pioneering Darwinian experiment, Sol Spiegelman *et al.* iteratively selected RNA molecules based on their ability to be replicated by Q bacteriophage RNA polymerase.^6^ Over the next two decades, such *in vitro* evolution experiments shifted towards more application-driven approaches, which is exemplified by the development of phage display.^7^ In phage display, an exogenous sequence is fused to a gene encoding a minor coat protein of a filamentous phage, leading the assembled viral particles to display the extra amino acids. A set of phages with different fused peptides could then be subjected to affinity purification, against desired binding partners, to obtain variants with high affinity towards them.

**Fig. 1:**
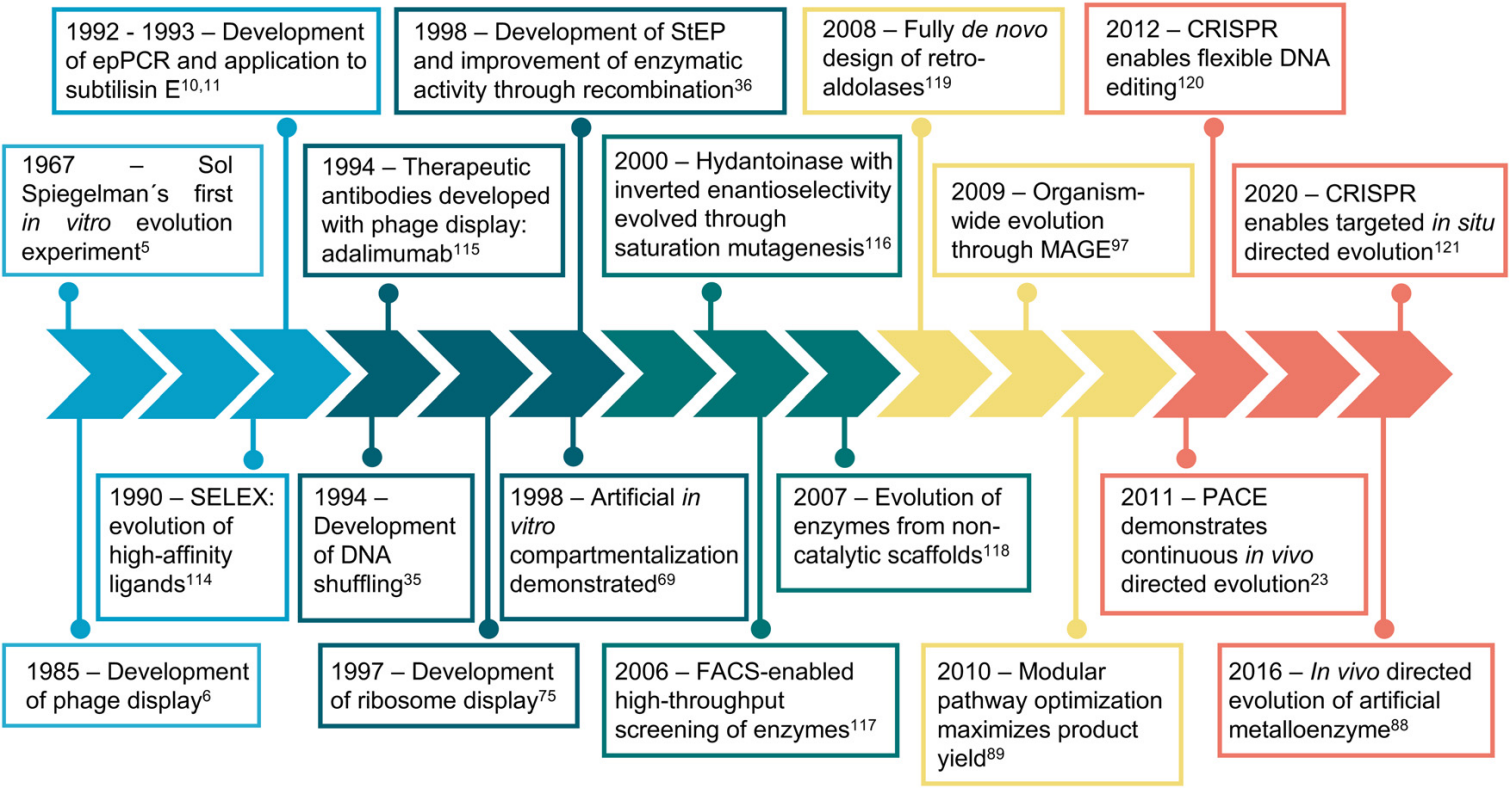
Timeline of directed evolution major developments. Representative examples of some of the most relevant developments and achievements in directed evolution are shown.

**Fig. 2:**
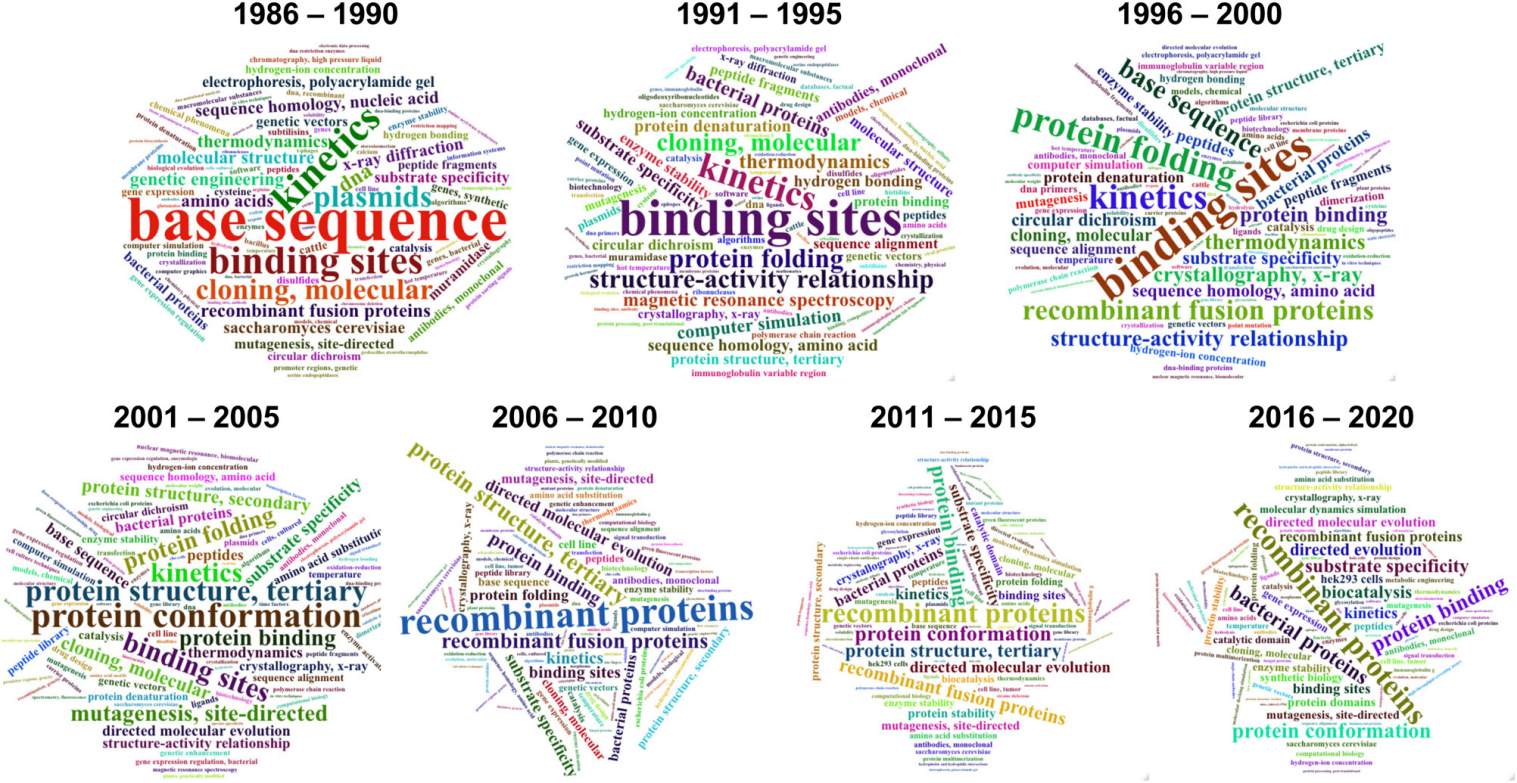
Evolution of keywords associated to directed evolution articles. Wordclouds of the keywords associated to directed evolution articles since 1985 are shown. Articles were grouped in blocks of five years. The wordclouds reveal a change over time of the focus and scope of directed evolution, with the most recent decade showing a larger variety of targeted properties and biomolecules. Data was retrieved, analysed and visualised with R.

During the last 30 years, directed evolution approaches have diversified and shifted their focus towards more complex and varied properties and biomolecules (Fig. 1). This can be easily visualized by analyzing the frequency of keywords associated with directed evolution papers (Fig. 2). Our analysis suggests that from the early days of directed evolution and until the mid 2000s, directed evolution focused mostly on altering binding sites and improving enzyme kinetic parameters, with a special emphasis on the analysis of nucleotide and amino acid sequences when choosing the approach to take. At the beginning of the 21st century, protein structure and conformation became a major point of interest in directed evolution studies, probably due to the increasing availability of macromolecular structures as a consequence of the improvement and accessibility of structural biology techniques. Indeed, in the period 1995–2000, “structure–activity relationship” was one of the top keywords found in directed evolution papers. Such keywords ranked considerably lower from 2005, probably due to the realization that the mechanisms through which structure determines function in biological macromolecules cannot be easily unraveled. During the last decade, the variety of targeted properties has quickly increased (including a larger proportion of studies aiming to alter substrate specificity). The range of organisms employed in such studies has also been expanded, as demonstrated by the presence of keywords such as “HEK293 cells” or “cell line”. Interestingly, a persistent interest in recombinant proteins seems to have been sustained during the same period.

The large expansion of the scope of directed evolution is tightly linked to the ample variety of developed techniques that allow researchers to tackle more efficiently the two main steps of the process of artificial evolution of biomolecules (Table 1). The first step consists in generating enough genetic diversity in a given parental sequence to cover the sequence-function space to be sampled. The resulting set of sequences, or library, often includes a majority of variants without the desired property or improvement. In a second step, the individual genotypes must be linked to the individual phenotypes^8^ to allow the variants of interest to be identified and isolated from the library. In this review, we aim to provide an overview of the different available methodologies, presenting the underlying principles, advantages and disadvantages of each one in order to facilitate readers to make an informed choice when determining the most appropriate techniques for a particular application of directed evolution.

**Table 1:** Summary of techniques frequently applied in directed evolution

	Technique	Purpose	Advantages	Disadvantages	Application examples
Mutagenesis	Error-prone PCR and error-prone RCA	Insertion of point mutations across whole sequence	• Easy to perform	• Reduced sampling of mutagenesis space	Subtilisin E^12^
			• Does not require prior knowledge about key positions	• Mutagenesis bias	Glycolyl-CoA carboxylase^124^
	RAISE	Insertion of random short insertions and deletions	• Enables random indels across sequence	• Indels limited to few nucleotides	
				• Frameshifts introduced	β-Lactamase^17^
	TRINS	Insertion of random tandem repeats	• Mimics duplications that occur in natural evolution	• Frameshifts introduced	β-Lactamase^18^
	Mini-mu based techniques	Random insertion or deletion of one or multiple codons	• Conservation of reading frame		Arylesterase^18^
			• Modification of transposon enables customization of mutations	• Additional steps of DNA manipulation required	GFP^125^
	Mutator strains	*In vivo* random mutagenesis	• Simple system	• Biased and uncontrolled mutagenesis spectrum	Vitamin K epoxide reductase^126^
				• Mutagenesis not restricted to target	Cells resistant to DMF^127^
	Orthogonal systems based on DNA Pol I, pGLK1/2, Ty1, T7RNAP and CRISPR	*In vivo* random mutagenesis	• Mutagenesis restricted to target sequence	• Mutation frequency relatively low	β-Lactamase^28^
			• Could be coupled to *in vivo* selection	• Limitations on size of target sequence	Dihydrofolate reductase^128^
					Orotidine-5′-phosphate decarboxylase^31^
	DNA shuffling	Random sequence recombination	• Recombination advantages	• High homology between parental sequences required	Thymidine kinase^129^
					Non-canonical esterase^130^
	StEP	Random sequence recombination	• Recombination advantages	• High homology between parental sequences required	Photoswitchable fluorescent protein^131^
			• Easy to perform		Synthetic antibodies^39^
	RACHITT	Random sequence recombination	• Increased crossover frequency		Monooxygenase^40^
			• Parental sequences removed from library	• High homology between parental sequences required	DNA polymerase^132^

Mutagenesis	ITCHY and SCRATCHY	Random recombination of any two sequences	• No homology between sequences required	• Gene length and reading frame not preserved	Alcohol dehydrogenase^41^
				• Recombination at sites not structurally related	Deoxyribonucleoside kinase^133^
				• Single crossover per variant (solved in SCRATCHY)	
	SHIPREC	Random recombination of any two sequences	• No homology between sequences required	• Single crossover per variant	
			• Crossovers at structurally-related sites	• Reading frame not preserved	Cytochrome P450^42^
	SISDC	Recombination of a few sequences at specific sites	• No homology between sequences required	• Limited number of potential crossover points	
			• Crossover points can be chosen	• Additional steps of DNA manipulation required	β-Lactamase^44^
	MORPHING	*In vivo* generation of recombination libraries	• Can be coupled to *in vivo* selection techniques	• Crossovers only occur at overlapping regions	Peroxidase^45^
					Aryl alcohol oxidase^134^
	Site-saturation mutagenesis	Focused mutagenesis of specific positions	• In-depth exploration of mutagenesis at chosen positions	• Only a few positions mutated	Widely applied to enzyme evolution^56,57^
			• Possibility to incorporate previous information for efficient mutagenesis	• Libraries can easily become very large	
			• Iterative cycles and smart libraries can reduce library sizes		
	StLois	Sequential extension of loops	• Insertions performed at sites less likely to result in non-functional variants		
			• Reasonable library sizes due to sequential extension of loops	• Limited number of insertions in each extension cycle	Cumene dioxygenase^64^

Identification of variants	Colorimetric/fluorimetric analysis of colonies/cultures	Screening of variants	*•* Fast and easy to perform	*•* Limited to biomolecules exhibiting appropriate spectral properties	Fluorescent proteins^57,58^
	Plate-based automated enzymatic assays	Screening of variants	• Automation has increased throughput	• Throughput remains limited compared to other methods, especially if substrate or product do not have characteristic spectral or fluorescent properties	
			• Surrogate substrates expand scope	• Results with surrogate substrates do not always replicate with original ones	Lipase^135^
			• Coupling to GC/HPLC enables analysis of enantiomers		Laccase^136^
	FACS-based methods	Screening of variants	• High throughput	• Evolved property must be linked to a change in fluorescence	Sortase^81^
			• Product entrapment expands application scope		Cre recombinase^82^
			• Similar techniques can be applied with *in vitro* compartmentalization		β-galactosidase^83^
	MS-based methods	Screening of variants	• High throughput	• Less widely-available equipment required	Fatty acid synthase^77^
			• Does not rely on specific properties of substrates	• For MALDI-based methods, requirement of immobilization on matrix	Cytochrome P411^78^
					Cyclodipeptide synthase^79^
	Display techniques	Selection of variants	• High throughput	• Limited to selection of biomolecules with specific binding properties	Antibodies^87^
					Fbs1 glycan-binding protein^90^
					RNA-binding peptides^137^
					Random sequence ATP-binding proteins^138^
	QUEST	Selection of variants	• High throughput	• Limited scope due to substrate/ligand constraints	Scytalone dehydratase^101^
					Arabinose isomerase^139^
	Cofactor regeneration coupling	Selection of variants	• High throughput		Alcohol dehydrogenase^109^
			• Applicable to wide range of small molecule biocatalysts and properties	• An indirect link to NAD-related activities must be established	Imine reductase^109^
					Nitrorreductase^109^
					Isopropanol pathway^109^
	*In vitro* compartmentalized self-replication	Selection of variants	• High throughput	• Limited to activities that can be linked to replication or transcription of its coding sequence	DNA polymerase^104^
			• Bypasses library transformation		

## Generation of a library of variants

The rate of natural mutation is usually insufficient for generating the genetic diversity required for laboratory directed evolution. For example, the mutation rate of wild-type *E. coli* is approximately 1 × 10^−3^ mutations per genome per generation, or 2.2 × 10^−10^ mutations per base pair per generation.^9^ With such a low mutation rate, over 100 000 generations would be required on average to obtain a single point mutation in a target gene of 1000 base pairs.^10^ Therefore, it is necessary to artificially enhance genetic diversification to increase the sampling of mutations. A plethora of techniques to achieve this are now available, each having their own advantages and drawbacks. They can be broadly classified into random and rational mutagenesis, although it is often found that a given technique combines aspects from both types of approaches.

### Random mutagenesis

In random mutagenesis approaches, no specific sequence positions are targeted. Such techniques are particularly useful for directed evolution of proteins for which there is not enough structure–function information available to determine which residues to diversify, or when the property to be evolved cannot be easily attributed to a few specific positions. For instance, enhanced stability under extreme conditions such as high temperature or organic solvents.

#### *In vitro* techniques

**Fig. 3:**
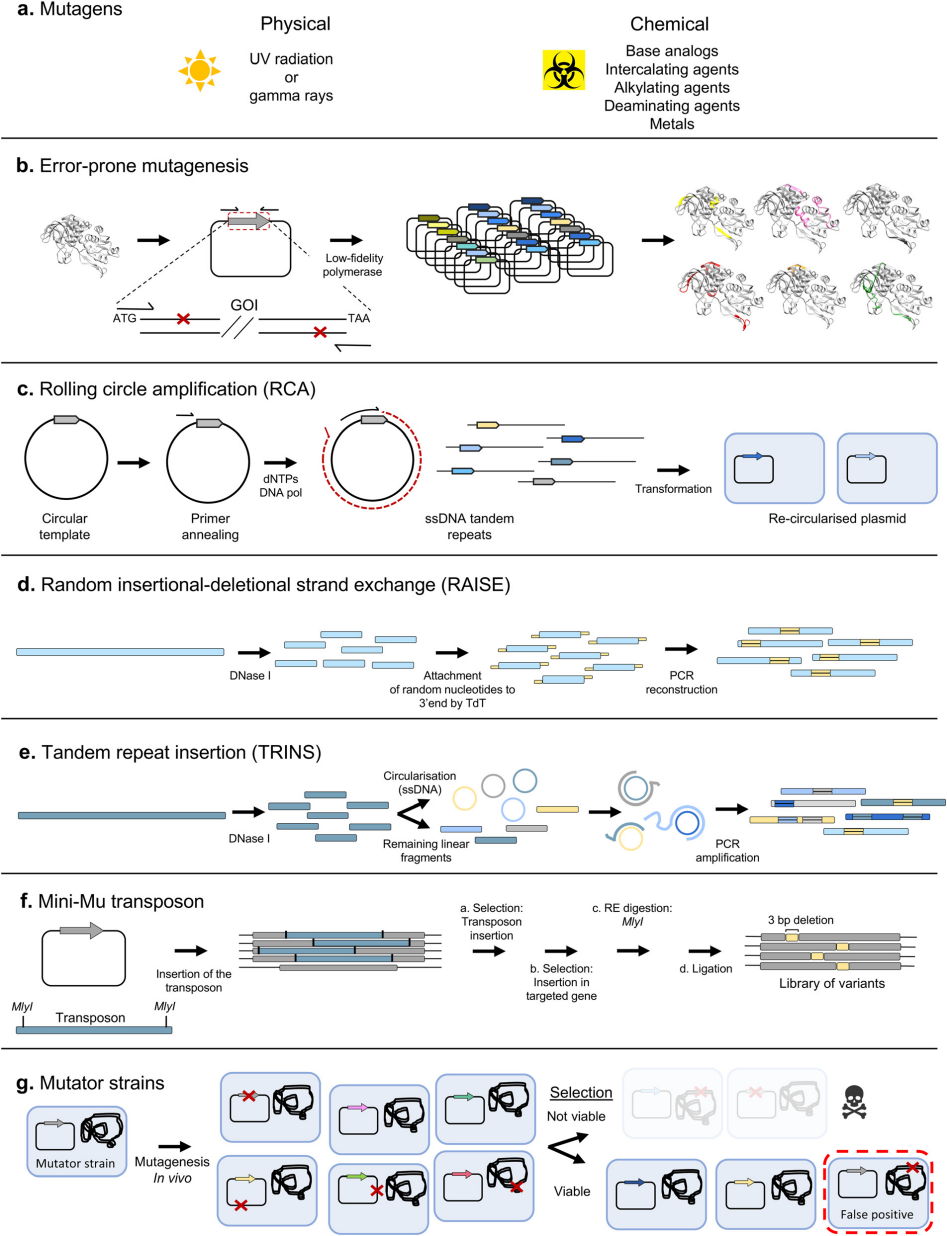
Random mutagenesis techniques. Some of the most widely applied random mutagenesis approaches are represented. (a) Chemical and physical mutagens were the basis of many genome-wide screening experiments, but have not been extensively used in directed evolution. (b) In error-prone PCR, random mutations are introduced by a low fidelity polymerase, resulting in linear DNA fragments with point mutations, which generally require further manipulation to be inserted into an appropriate vector. (c) RCA based methods bypass the need for further manipulation, since the products can be automatically recircularized by the host cells. (d) RAISE was one of the first methodologies designed to introduce random insertions in the sequence to be mutated. Random small extensions are attached to digested fragments of the parental sequence, and full-length genes are reconstructed through PCR. (e) TRINS aims to generate repeats of random short fragments of the parental sequence. First, the parental sequence is digested with DNase I. Part of the obtained fragments are circularized and mixed with the remaining linear fragments. An assembly PCR reaction is then performed. When a linear fragment anneals with a circular fragment, a reaction similar to RCA takes place, leading to the replication of multiple copies of the region corresponding to the circularized fragment. (f) Several mutagenesis techniques based on the mini-Mu transposon have been devised. Such techniques allow the generation of random insertions or even deletions while preserving the appropriate reading frame. (g) Mutator strains were the first tool enabling *in vivo* random mutagenesis. However, the increased mutagenesis rate applies to the whole genetic material, and not only to the sequence of interest. More sophisticated approaches where only the gene of interest is targeted have been developed. GOI: gene of interest.

Some of the earliest mutagenesis techniques were based on the use of chemical or physical mutagens to introduce random mutations in the sequence of interest^11^ (Fig. 3a). However, these have been superseded by other methods for directed evolution due to their strong bias towards certain mutations, and the need to perform further manipulations to obtain double-stranded mutagenised DNA.

Error-prone PCR (epPCR)^12^ is one of the most widely used techniques for random mutagenesis (Fig. 3b). In epPCR, a PCR is performed with low-fidelity polymerases (such as Taq polymerase) and altered reaction conditions (including Mn^2+^ and increased Mg^2+^ ions, and using unequal concentrations of the different deoxyribonucleotides) to increase the error frequency. In a landmark study, Chen and Arnold demonstrated the potential of the technique by alternating rounds of epPCR and screening to obtain a variant of subtilisin E enzyme, with increased activity in the presence of 60% dimethylformamide.^13^

Approaches based on epPCR require fine-tuning of the mutation rate, which must be neither too high nor too low (this varies by application, but a rule of thumb is 1 mutation per kb per generation). Achieving an optimal mutation rate is crucial to obtain a library with a good density of functional variants, since an excessive amount of mutations can easily lead to a prevalence of deleterious effects, depleting the library from variants of interest. Approaches to alleviate the load of non-functional variants have been devised, such as the construction of neutral drift libraries.^14^ In such methodology, variants are pre-screened to identify those that maintain wild-type activity, which are then pooled to create a library of variants without deleterious effects, used as the starting point for additional rounds of selection for the actual target property. In addition, transformation into a host strain is often the limiting factor in library size, leading to the loss of some variants. It can therefore be advantageous to amplify the library prior to transformation, for example using plasmid rolling circle amplification (RCA)^15^ (Fig. 3c). Thus, in 2004, a simple protocol for error-prone RCA (epRCA) was developed, where the mutagenesis rate is enhanced through the addition of MnCl_2_ to the reaction, and by reducing the concentration of the DNA template.^16^

Both epPCR and epRCA only introduce substitutions in the sequences subjected to mutagenesis. However, insertions and deletions (indels) also play an important role in natural genetic diversification, since they can alter the backbone of the encoded proteins in a way not achievable simply by point mutation.^17,18^ Several methodologies have been developed for introducing indels into gene libraries. Initial efforts focused on the introduction of random insertions and deletions, such as the RAISE method, where 3′-terminal deoxynucleotidyl transferase introduces random extensions^19^ (Fig. 3d).

Insertions by duplication are another of the most frequent naturally occurring indels, with some estimates placing their frequency as high as two thirds of all insertions observed in natural genomes.^20^ TRINS was developed as a method to mimic such insertions *in vitro*, resulting in the formation of tandem repeats of segments of the sequence of interest through assembly PCR with random linear and circularised fragments^20^ (Fig. 3e).

Nevertheless, such techniques have the drawback of generating variants with frameshifts, which usually lead to loss of function. More refined indels can be achieved with modified versions of the mini-Mu transposon, a transposable element which can be easily inserted at random points of a DNA sequence by treatment with the MuA transposase.^21^ Jones modified the mini-Mu transposon to allow the removal of single codons at insertion sites after treatment with MlyI and ligation^22^ (Fig. 3f).

TRIAD uses a modified Mu protocol to insert or delete up to 3 codons.^18^ In it, an engineered transposon is inserted at random points, and then removed through treatment with MlyI. Multiple cassettes can then be inserted through ligation at the generated restriction sites. The deletion cassettes are designed such that their removal with a final treatment with the appropriate restriction enzyme also leads to the deletion of a fixed number of adjacent nucleotides. On the other hand, insertion cassettes include degenerate NNN codons, which remain in the sequence of interest even after removal of the rest of the cassette. More recently, a DNA assembly platform to introduce customizable insertions has been devised,^23^ based on cycles of endonuclease treatment and ligation of DNA sequences with the desired inserts.

#### *In vivo* techniques

While *in vitro* mutagenesis techniques enable a relatively good control over the mutation rate and spectrum, they also suffer from inherent bottlenecks, such as the limitation on the library size imposed by the transformation efficiency and the requirement of manipulating the genetic material. *In vivo* approaches try to bypass these limitations by taking advantage of the cell machinery to directly perform mutagenesis within the host cells. This also allows the coupling of mutation and screening or selection cycles, enabling more efficient automated workflows, including modern continuous directed evolution^24^ (Fig. 8).

The first *in vivo* mutagenesis systems were mutator strains, such as the XL1-Red strain,^25^ where inactivation of DNA repair pathways results in increased mutation rates (Fig. 3g). While such hypermutator strains provide a simple *in vivo* mutagenesis system, they present major drawbacks, including a relatively low and hard-to-control mutagenesis rate, biased mutagenesis spectrum, and the indiscriminate mutagenesis of the genome and other sequences outside the gene of interest. This causes a series of undesirable side effects, such as slower growth, reduced transformation efficiencies, and loss of successful variants. More recent random mutagenesis approaches have improved this by having inducible mutator plasmids of different strengths^26^ and have overcome genome background mutagenesis by using evolving phage that continuously infect virgin cells containing mutator plasmids.^27^

Despite these advances, the ‘Holy Grail’ in directed evolution is to perform targeted mutagenesis of the gene of interest. An early example is the two-plasmid system based on error-prone DNA polymerase I (Pol I), where Pol I introduces mutations in sequences of up to 3000 bp.^28^ A similar system for *in vivo* mutagenesis in yeast has also been devised, termed OrthoRep, based on the pGKL1/2 plasmid system of *Kluveromyces lactis*, where mutations are introduced by error-prone terminal-protein DNA polymerase 1.^29,30^ Other *in vivo* systems have expanded the idea to use enzymes other than DNA-dependent DNA polymerases as the main source of mutagenesis. An example is the Ty1 retrotransposon-based *in vivo* mutagenesis system for yeasts, based on the high error propensity of its encoded retrotranscriptase,^31^ or the MutaT7 system, based on chimeric protein fusing T7 RNA polymerase and a nucleobase deaminase, responsible for the introduction of mutations.^32^

More recently, there have been advances in targeted mutation based on CRISPR technology, such a EvolvR, which allows targeted mutation rates that are up to ∼8-million-fold greater than rates seen in wild-type cells, and editing windows with lengths of up to 350 nucleotides.^33^ These techniques promise a step-change for *in vivo* directed evolution. Nevertheless, despite these recent advances, it should be noted that *in vivo* mutagenesis is in practice rarely applied to the evolution of biocatalysts acting on small molecule substrates. This is due to the fact that there is not yet a generalized method to assess the fitness of the generated variants *in vivo*, although instances of successful directed evolution of novel enzymes acting on small-molecule substrates through *in vivo* mutagenesis coupled to selection exist, as later discussed. The lack of such a general method that enables the coupling of the different properties of a wide range of substrates or products to an increased survival rate or an easily screenable indicator, makes *in vitro* and rational mutagenesis the current preferred choice for the evolution of enzymes acting on small molecules.

### Recombination techniques

Recombination is one of the most powerful mechanisms in nature to generate the genetic diversification required for evolution.^34,35^ Correspondingly, a large variety of recombination-based techniques have been developed for directed evolution. They differ greatly from random mutagenesis techniques in the fact that they produce libraries of combinatorial variants where segments from several functional biomolecules are combined. In principle, libraries generated through recombination methods possess a higher percentage of functional variants, avoid the introduction of stop codons, and can lead to the discovery of epistatic mutations.^36^

**Fig. 4:**
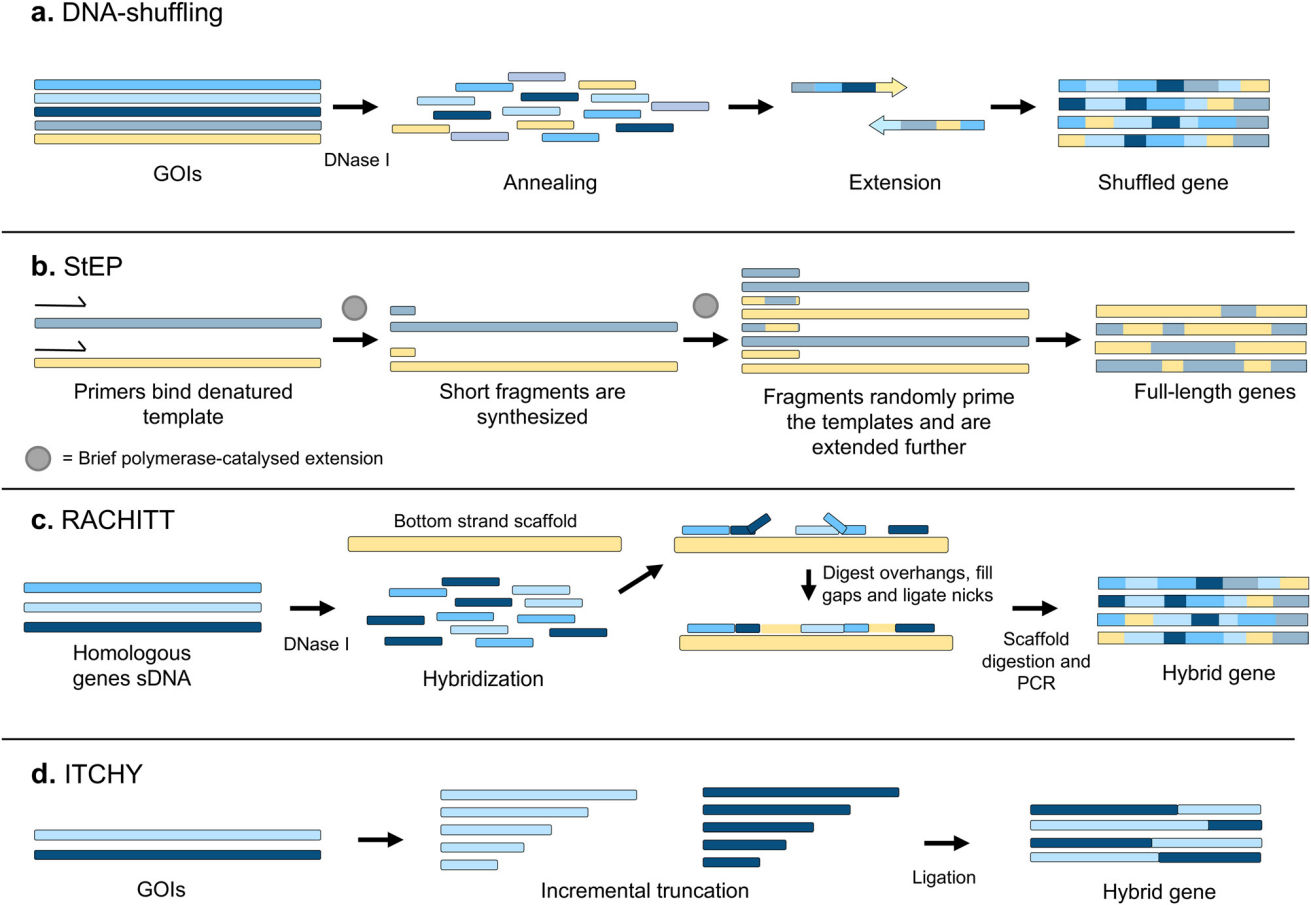
Recombination-based mutagenesis techniques. A set of some of the main mutagenesis techniques based on recombination is displayed. (a) DNA shuffling was the first recombination-based *in vitro* mutagenesis technique to be developed. A set of homologous sequences is treated with DNase I, and the resulting mix of fragments is used to reassemble full-length sequences through self-priming PCR. (b) In StEP, a set of homologous sequences is used as templates for a series of cycles of annealing with primers and extension of the primers by a DNA polymerase. In each cycle, the growing primers can anneal with a different template, resulting in chimeric full-length sequences. (c) RACHITT requires less homology between parental sequences than DNA shuffling and StEP. A set of fragments obtained by treatment with DNase I of the complementary strands of the sequences to be recombined is hybridized to a single-stranded copy of one of the parental sequences. After digesting overhangs, filling the gaps and ligating the nicks, the scaffold strands are digested, and double-stranded chimeric sequences are obtained by means of PCR. (d) ITCHY decreases even further the sequence homology requirements, but it is limited to a single crossover per variant. Exonuclease III is used to incrementally truncate one of the parental genes from its 3′ end, and the other one from its 5′ end. Then, random-length fragments of each gene are ligated. GOI: gene of interest.

The first recombination-based technique for library generation was DNA shuffling.^37^ A pool of closely related sequences is fragmented by DNase I treatment, and full-length sequences are reassembled through self-priming PCR, combining different parental sequences (Fig. 4a). Alternatively, StEP (Staggered Expansion Process) provides a technically simpler approach where the need to fragment the parental sequences with DNase I is bypassed.^38^ StEP consists of repeated cycles of denaturation of the templates and very short annealing and extension by DNA polymerase steps, performed until combined full-length products are obtained (Fig. 4b). Typically, StEP is applied to a pool of sequences generated by another mutagenesis technique, to further increase genetic variability and has proven to be useful for evolving synthetic antibodies with improved affinities.^39^

However, both DNA shuffling and StEP require a high degree of homology between the different parental sequences to be recombined. This makes them unsuitable for the recombination of distantly related sequences, which would be very useful for recombining homologous enzymes, for example. Several strategies have therefore been developed to reduce the required degree of homology.

RACHITT (Random Chimeragenesis on Transient Templates) employs a single strand of one of the sequences to be recombined as a scaffolding template, to which fragments of the other sequences are hybridised and ligated^40^ (Fig. 4c). Others went even further, seeking to develop techniques that completely removed any requirement for homology between the sequences. The first methodologies allowed only two genes to be recombined, such as ITCHY (Incremental Truncation for the Creation of Hybrid enzymes, Fig. 4d)^41^ and SHIPREC (Sequence Homology-Independent Protein Recombination),^42^ both of which combine 3′ and 5′ fragments of two genes. Further variation can be introduced with the SCRATCHY protocol,^43^ where variants from an ITCHY library are further recombined by DNA shuffling. Other methods, such as SISDC (Sequence Independent Site Directed Chimeragenesis),^44^ bypass the need for sequence homology by pre-establishing the crossover points in the parental sequences.

More recently, the focus of novel recombination methods has shifted towards the *in vivo* generation of libraries, allowing a direct coupling to selection techniques. One of the first such approaches was MORPHING (Mutagenic Organized Recombination Process by Homologous *in vivo* Grouping), which takes advantage of the naturally high recombination frequency of *Saccharomyces cerevisiae* to assemble full-length variants of a gene from sets of overlapping fragments.^45^

### Rational mutagenesis

In contrast to random mutagenesis approaches, rational mutagenesis focuses on mutating only a limited number of positions in the target sequence, which must be determined based on prior knowledge. The latter includes structures, multiple sequence alignments, biochemical data and computer-based predictions.

**Fig. 5:**
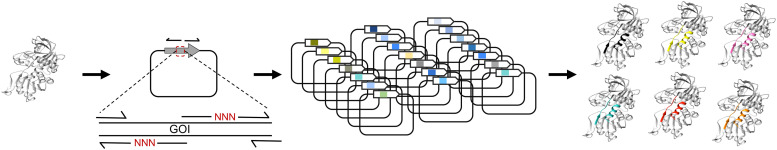
Site-saturation and site-directed mutagenesis. Site-saturation mutagenesis allows the introduction of a large range of point-mutations at specific sites, while site-directed mutagenesis introduces a specific set of mutations. In both cases, mutagenic primers, which contain mismatches with the parental sequence in the positions to be mutated, are used for PCR reactions with the parental sequence as the template. The amplification products are the mutated variants. In the case of site-directed mutagenesis, primers carrying specific mutations are used. For site-saturation mutagenesis, degenerated primers containing a range of possible mutations are employed. GOI: gene of interest.

Site-saturation mutagenesis (SSM) enables fast and efficient generation of libraries where all possible substitutions of the chosen positions are included. Several different strategies have been developed to obtain libraries with saturated positions, but most rely on the use of mutagenic primers that randomise chosen positions in PCR (typically using the base N in oligonucleotides, where N is an equimolar mixture of A, C, G and T). One of the first developed protocols was overlap extension PCR (OE-PCR),^46^ where two sequential steps of amplification are performed. In the first step, overlapping primers, degenerate on the position(s) to be mutated, result in two overlapping segments of sequence containing the mutation(s). The full-length sequence is reconstituted in a second amplification step where primers annealing with both ends of the sequence are used (Fig. 5).

While this protocol can be easily used to saturate one or a few proximal codons (close enough to be included in one or two primers), more specialised strategies are required in order to generate libraries where several distant sites are randomised. One possibility is to use several pairs of mutagenic degenerate primers covering the full sequence, such that both the reverse primer for a given segment and the forward primer for the next segment overlap partially and cover the same degenerate codon. The resulting products are separated by agarose (MOE-PCR) or polyacrylamide gels (POEP), and full-length sequences are assembled by means of a second PCR with flanking primers.^47,48^ It is also possible to anneal all mutagenic primers simultaneously to the parental sequence, extend them with T4 DNA polymerase and perform a PCR to obtain the library. However, this can be difficult to achieve if the thermodynamic parameters of each set of primers differ. OmniChange was developed as an alternative to saturate up to five independent codons in a sequence-independent manner by using primers with phosphorothiodiester bonds.^49^

In addition to the mutagenesis protocol, another key parameter that needs to be decided before undertaking SSM is the degeneracy scheme. It is possible to vary the target codons to NNN (where N represents A, G, T or C) to obtain libraries where all possible 64 triplets are present. However, since some amino acids are encoded by more possible codons than others, not all substitutions become equally frequent at the protein level. Furthermore, premature stop codons are introduced, leading to a background of variants with loss of function. Moreover, if a large number of codons is targeted, the library size can easily become excessive.

An NNK scheme (where K represents either G or T) reduces the library size by half and includes only one possible stop codon instead of three, while still allowing for all 20 amino acids to be encoded in the resulting triplets.^50^ More restrictive degeneration strategies can be implemented to completely remove stop codons and balance better the representation of amino acids with different chemical properties, albeit at the cost of not encoding all 20 amino acids^50,51^ (Table 1). A potential workaround is the usage of mixtures of primers with restricted degeneration schemes that encode, as an ensemble, all possible substitutions with only one possible codon per amino acid. This allows the generation of “small-intelligent” libraries, where stop codons and rare codons can be completely removed. DC-Analyzer was developed as a software tool to assist in the design of such libraries, with the possibility of generating libraries including only polar or hydrophobic residues.^52^ Nevertheless, the approach cannot be easily applied to cases where more than two sites must be randomised. Library size can be further reduced by randomizing only small groups of spatially-close residues located, for example, at the active site, as in the combinatorial active-site saturation test (CAST).^53^ ISM^54^ extended this methodology by employing the best resulting variants as templates for iterative cycles of randomization of additional clusters of residues, proving to be particularly successful for evolving enzymes with altered substrate specificity. Based on the same concept, FRISM^55^ was devised to iterate over multiple sites predicted to be key for the evolved property, but introducing only a few rationally determined mutations in a given position at each cycle, instead of generating libraries. Such approaches have achieved the highest success rate when evolving enzymes with altered substrate or product specificity (especially when performed iteratively), since they allow for mutagenesis to be focused around the active site and substrate binding pocket.^56,57^ Nevertheless, it is necessary to keep in mind that the assumptions that must often be made in order to reduce the library size to reasonable numbers, together with the fact that frequently not all possible combinations of mutations are assessed (which prevents the discovery of synergistic effects), risk removing optimal variants from the sequence space, making the evolution of enzymes with substrate specificity altered at will a non-trivial process (Fig. 8).

Some alternative attempts at improving the standard SSM protocols tackle generating optimal degenerate libraries from the point of view of the mutagenic primer synthesis. A near optimal solution was provided by the use of mixtures of 20 trinucleotide phosphoramidites, each encoding a different amino acid.^58,59^ Although this procedure allows for the elimination of codon bias and termination codons, the high cost of the technique has prevented its widespread application. Gaytán *et al.* developed a system to produce degenerate mutagenic primers in a cost-effective way, where stop and redundant codons are eliminated, named TrimerDimer.^60^ The method is based on the use of modified di and trinucleotides that are sequentially combined during solid phase synthesis to yield 20 random codons per position encoding all 20 amino acids and no stop codons. While TrimerDimer provides an attractive way of obtaining well-balanced libraries without variants with premature stop codons, its technical and equipment requirements do not make it readily available to all standard molecular biology laboratories. Such libraries can also be synthesized through commercially available services, at increasingly lower costs, which is becoming a more prominent route to obtain libraries. However, it should be noted that it is still not possible to synthesize some particularly complex library designs, such as those having multiple clusters of degenerate codons spread along the sequence or highly repeated sequences. Protocols such as Combinatorial Codon Mutagenesis (CCM) provide an interesting alternative for these cases.^61^ In short, iterative rounds of fragment PCR using mutagenic primers enabling codon degeneration at different target points, and joining PCR to generate full-length assemblies are performed. This approach, an example of a combination of rational and random mutagenesis, does not require specialized reagents, and therefore offers a cost-effective way to obtain libraries with a high number of saturated sites, including non-contiguous codons.

Computational tools can also provide valuable information to guide library generation. For example, CSR-SALAD employs structural information to predict a set of residues likely to have a key role in determining the nicotinamide cofactor preference of NAD(P)-dependent oxidoreductases.^62^ However, it relies on the availability of an accurate model of the structure of the target protein. While experimentally solved structures are not always available, the recent developments of accurate machine learning-based techniques, such as AlphaFold 2,^2^ could largely facilitate the choice of key residues to be targeted during mutagenesis, expanding the number of macromolecules that can be subjected to rational directed evolution (Fig. 8). The availability of a large number of accurate structural predictions also facilitates the adoption of mixed rational-random mutagenesis approaches. For example, the Stepwise Loop Insertion Strategy (StLois) aims to perform multiple rounds of successive insertions of random pairs of residues coupled to screening to identify the best variants. Insertions are introduced at loops expected to tolerate variability in proximity to active sites and are identified through structural alignment of multiple related proteins. Performing stepwise insertions of one or two residues only allows to keep reasonable library sizes at each round, and has been shown to be an effective tool for engineering biocatalysts.^63,64^

Machine learning (ML) has also been applied to guide library generation by modelling the fitness landscape incorporating multiple data sources of tested variants, achieving success at even evolving novel enantiospecific enzymes.^65,66^ One of the main difficulties found when applying ML to directed evolution is the scarcity of labeled data, *i.e.*, biological sequences with an associated measurement of the target property.^67^ One currently active research line aims to employ unlabeled sequence data to capture a set of underlying rules assumed to be followed by any functional protein, which can then be employed to generate a sort of compressed numerical representation of protein sequences (known as “embeddings”). Embeddings can then be provided to other models trained on the available labeled data, to expand the sequence space for which prediction of function is performed.^68,69^ Alternatively, training models on unlabeled data has been proven to be useful in predicting weather a given sequence is likely to be functional or not through zero-shot prediction, which allows to eliminate non-functional sequences from subsequent experimental efforts.^70^ It is also possible to use the learnt underlying sequence distribution to generate candidate sequences for novel functional variants with generative models such as generative adversarial networks.^71,72^ This approach has been shown to be successful at identifying a functional malate dehydrogenase differing as much as 106 point mutations from the original sequence, which would have been impossible to identify either experimentally or with traditional predictive models due to the large size of the involved sequence space.^72^

## Identification of the variants of interest

The main bottleneck of directed evolution is often the identification of the variants of interest. Typical libraries generated in modern directed evolution experiments can include many millions of variants, with the vast majority of sequences lacking the desired properties. Two main strategies have been developed to isolate successful variants: screening and selection.

Screening approaches aim to evaluate the target property across the library, leading to the identification of the best-performing variants. On the other hand, selection approaches link an improvement in the evolved property to a physical recovery of the corresponding coding sequence, or an increased survival rate.

### Screening techniques

**Fig. 6:**
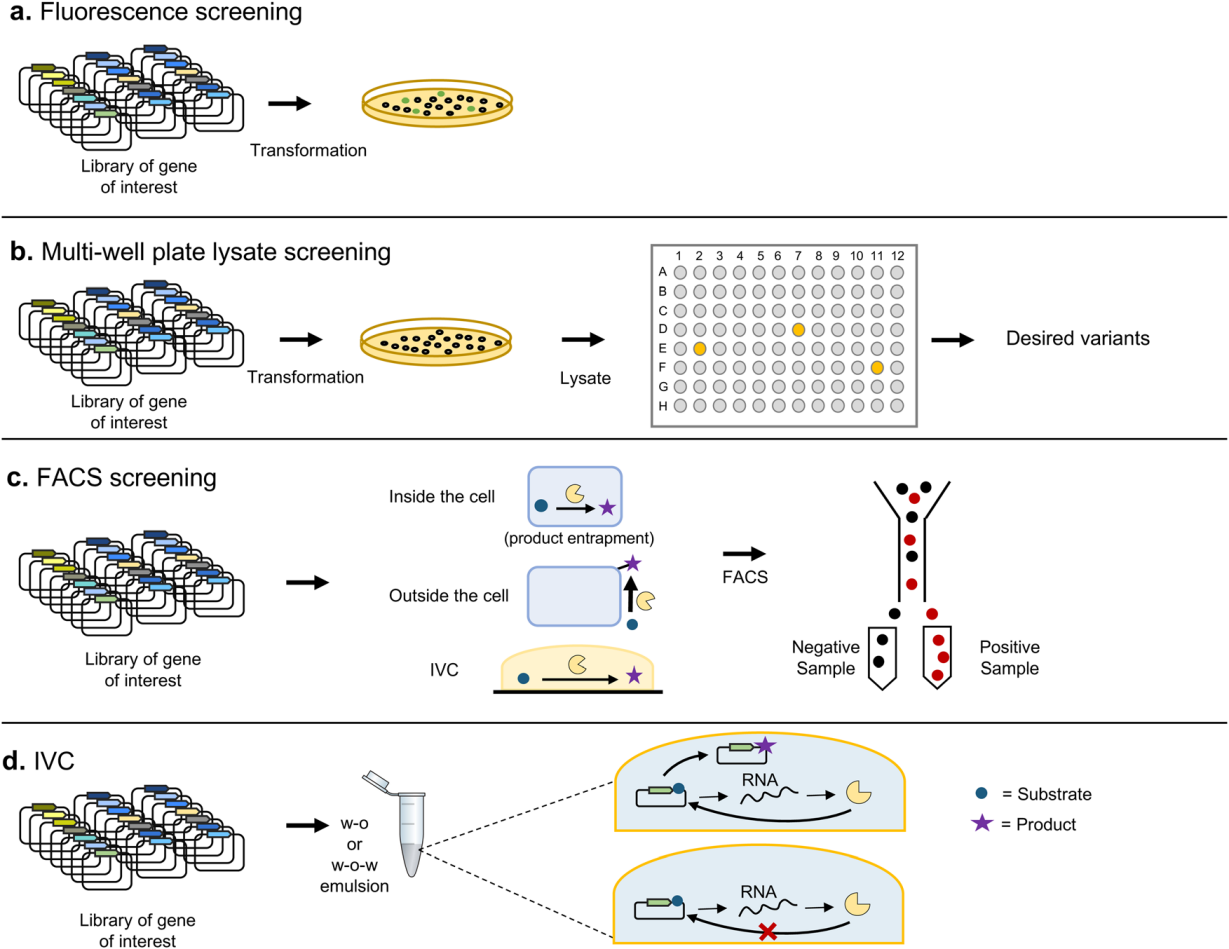
Screening techniques. Some of the most frequent screening techniques are depicted. (a) Variants of proteins that confer fluorescence can be screened by analysing with digital imaging techniques cultures in solid media. (b) For proteins whose activity can be linked to a colorimetric assay or to the generation of fluorescence, it is possible to automatically transfer individual colonies to liquid cultures by means of automated multi-well liquid culture devices. The liquid cultures or their lysates can then be screened by colorimetric or fluorescent-based assays. (c) FACS enables the physical separation of individual cells based on their fluorescence properties, allowing for a higher throughput and reduced material and physical requirements. However, it is limited to biomolecules whose activity can be linked to a change in fluorescence. (d) IVC techniques replace the compartmentalization provided by cells with artificial compartmentalization, most frequently provided by emulsions of water and oil. This allows to bypass the limitation imposed by transformation efficiency, but incompatibilities between the conditions required for transcription and translation and those required for the activity of the biomolecule of interest reduce its scope of application. IVC: *in vitro* compartmentalization; w–o: water-in-oil; w-o-w: water-in-oil-in-water.

The most basic screening techniques rely on spatially separating each variant and then assessing their activities individually. This can be achieved by expressing the library of variants in a model organism, such as *E. coli*, and plating on solid media to isolate colonies corresponding to clones containing a single variant. In some cases, it is possible to perform colorimetric or fluorometric measurements directly on the colonies obtained, thanks to automated digital imaging techniques (Fig. 6a). It is also possible to transfer the colonies to multi-well liquid culture devices to perform further analysis with the liquid cultures themselves (or cell lysates) on microtiter plates. Robotic systems allow tracking thousands of such assays simultaneously (Fig. 6b).

Some biomolecules possess spectral properties that can be directly screened, such as fluorescent proteins. There are indeed multiple cases of successful identification of variants of GFP with more intense fluorescence,^73^ or altered absorption or emission spectra.^74^ However, most frequently it is the consumption of an enzyme substrate, or product, that is measured. When either substrate or product has a distinctive absorbance or fluorescence peak, it is possible to use the signal to evaluate enzymatic activity. Alternatively, variants can be assessed with a surrogate substrate exhibiting the desired spectral or fluorescent properties. However, the identified candidates of interest must then be reassessed with the original substrate to confirm activity. This is of special relevance when the target property is altered or improved enantiospecificity, since different substituents on the substrate can greatly affect such properties.^75^ Under such cases, automated chiral GC and HPLC offer a better solution for screening positive hits.

When a convenient property of the product of interest that can be easily assessed cannot be identified, methods based on mass spectrometry (MS) provide a powerful alternative. MS offers multiple advantages over other screening methods, such as its high sensitivity and specificity, as well as the absence of a requirement for specific spectroscopic properties in the target molecule (which also eliminates the need to create surrogate substrates). Furthermore, the main limitation of the technique, the requirement for a time-consuming separation step (typically performed by HPLC), has been considerably overcome with recent developments in automated and fast autosamplers, such as Agilent's RapidFire platform, which have greatly reduced the required time down to only a few seconds per sample while employing electrospray ionization (ESI) for sample preparation.^76^ Matrix-assisted laser desorption/ionization (MALDI) can also provide a very high throughput but requires immobilization of the targets onto a matrix. Therefore, this requires careful consideration of the chemistry of the target molecules to develop an appropriate immobilization method and matrix, although the effectiveness of the method for screening of novel biocatalysts has been demonstrated for a variety of cases.^77–79^

Despite automation, screening by physical separation in wells limits the number of variants that can be tested. This can be overcome by directly analyzing the different variants in bulk without previous separation, for example by fluorescence-activated cell sorting (FACS)^80^ (Fig. 6c). FACS allows the separation of individual cells by means of flow cytometry, based on fluorescent signals, allowing screening of up to 10^8^ variants in a few hours,^81^ as long as their activities can be linked to a change in fluorescence. For example, Santoro and Schultz developed a reporter where GFPuv would only be expressed upon recombination of two loxP sites. With such an arrangement they were able to select Cre recombinase variants with increased activity towards modified loxP sites.^82^

It is also possible to use a substrate that is converted by an enzyme of interest to a fluorescent product unable to leave the cell. After washing off the permeable substrate, cells containing an active variant can be identified through the fluorescence of the product. This methodology, known as product entrapment, has been applied to a variety of cases, including glycosyl-transferases, glutathione transferase and β-galactosidase.^83^ FACS has also been applied to screen protein–protein interactions by fusing one of the interactors to the yeast Aga2 surface receptor.^81^

There have also been attempts to replace the natural compartmentalization provided by cells with artificial *in vitro* compartmentalization (IVC) (Fig. 6d). IVC offers the advantage of bypassing the transformation of the library, which can limit its effective size. Artificial compartmentalization is most frequently achieved through water-in-oil and water-in-oil-in-water emulsions. The latter, combined with microfluidics, allow the processing of up to millions of droplets per second, providing the basis for high-throughput screening.

IVC was first applied to identify genes encoding the HaeIII DNA methyltransferase amongst a hundred-fold excess of genes encoding other enzymes, by exploiting the resistance to restriction enzyme cleavage introduced upon methylation.^84^ IVC can also be coupled to FACS, with the requirement that water-oil-water must be used in order for the flow cytometer to handle the emulsion. Tawfik and collaborators employed this approach to obtain variants of paraoxonase 1 with increased activity towards sarin-like nerve agents.^85^

More recently, microfluidic systems have also been combined with direct detection through ESI-MS to achieve a throughput of nearly 1 sample per s.^86^ In principle, such a system enables sorting of nanodroplets without the limitations imposed by fluorescence-based methods, and without the requirement to immobilize the samples onto a MALDI matrix.

### Selection techniques

Selection methods automatically discard all undesired variants and thus tend to allow a higher throughput than screening, but are usually less generally applicable to a broad range of biomolecules. Two main categories of approaches can be distinguished, depending on whether the activity of a variant of interest leads to a physical segregation of its encoding sequence, or to an increased survival rate of the host organism.

#### Display techniques

**Fig. 7:**
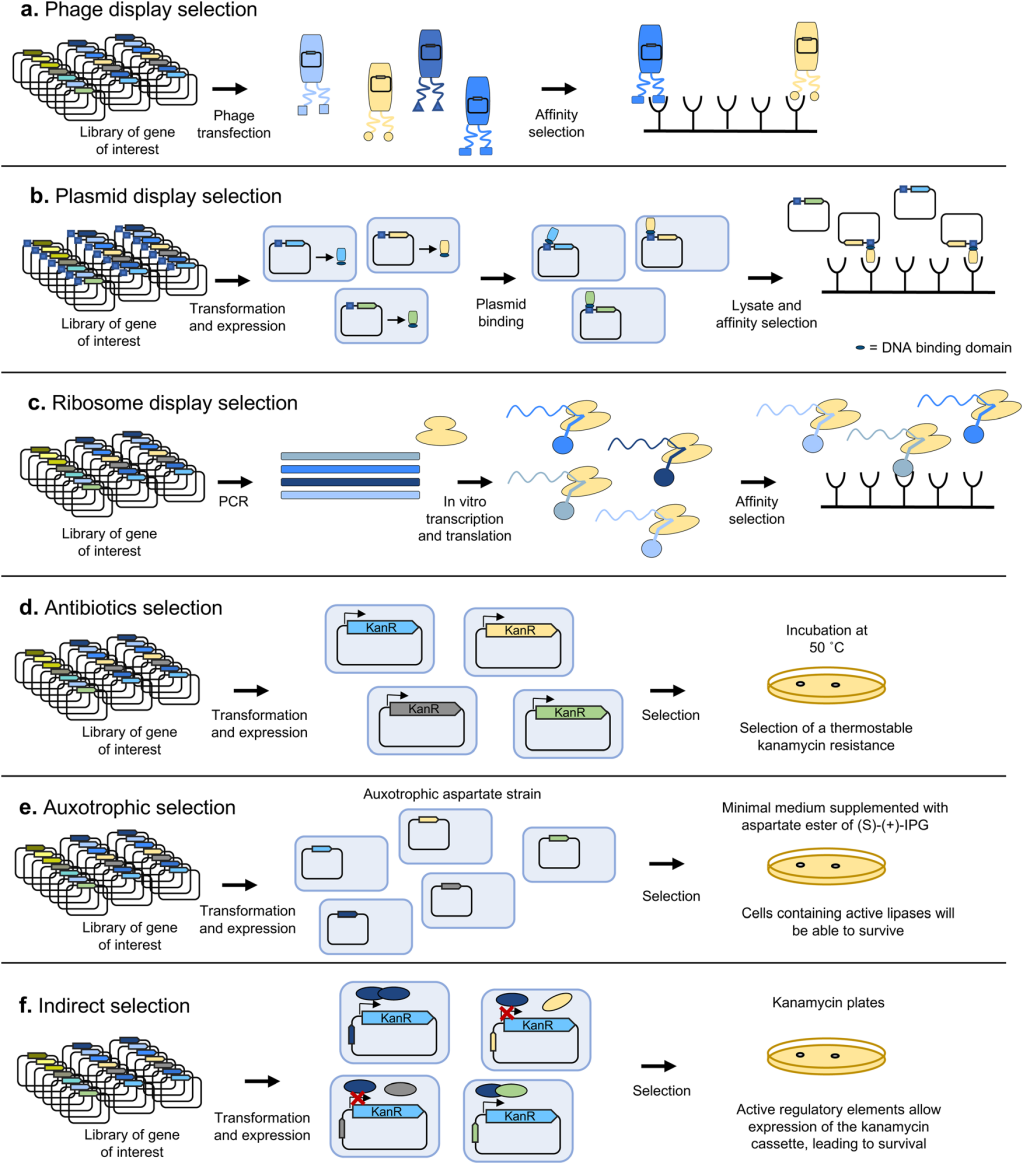
Selection techniques. Some of the most frequent selection techniques are represented. (a) In phage display, protein or peptide variants are exposed on the surface of phages, and selected based on their binding affinity to a target binding partner. (b) In plasmid display, a DNA-binding protein is fused to each variant. The encoding plasmid contains the target sequence for the DNA-binding protein. After lysing cells, variants can be selected based on their binding affinity to specific target interactors. Variant sequence can then be determined from the associated plasmid thanks to the linkage provided by the DNA-binding protein. (c) Ribosome display can be used to link protein variants to their corresponding mRNA. Translation is stopped by cooling on ice, and protein–ribosome–mRNA complexes are stabilized through the addition of magnesium, enabling affinity selection and amplification of the sequence of selected variants by treatment with reverse transcriptase, followed by PCR. (d) Antibiotic resistance selection is one of the most basic types of growth complementation selection techniques. Cells are transformed with a library of variants and grown in selective medium supplemented with a certain antibiotic. Only cells expressing a variant able to confer resistance for the added antibiotic and functional under the selection conditions (such as high temperature) will survive. (e) In auxotrophy-based selections, cells auxotrophic for a certain metabolite are grown in minimal medium without said metabolite but with a precursor that can yield the required compound upon transformation by a certain enzymatic activity. Cells are transformed with a library of variants, such that only those carrying a variant able to catalyse the conversion of the precursor will be able to survive. (f) In indirect growth-complementation based selection techniques, the activity of the gene of interest is not directly responsible for an increased survival rate. Instead, its activity (such as activation of transcription) leads to an increased expression or activity of the biomolecule directly responsible for it, such as an antibiotic resistance.

Display techniques rely on a physical connection between a nucleic acid sequence and the product it encodes, and are most frequently applied to obtain variants with improved binding to desired targets, with only a few exceptions of application to the evolution of catalysts available.^81^ The most common method employs the previously-described phage display technique to expose variants in the surface of phages which contain the gene encoding the corresponding variant (Fig. 7a). This has become one of the most powerful techniques to select peptides and antibody fragments with specific binding properties, leading to the development of numerous antibody-based pharmaceuticals which are in late-stage clinical trials or have already been approved.^87^ In addition to filamentous phages, other viruses can be used, such as the T7 phage for cytoplasmic proteins^88^ or retroviruses and other eukaryotic viruses for eukaryotic proteins.^89^

Another, conceptually simpler, approach is plasmid display (Fig. 7b), where each encoded variant is fused to a DNA-binding protein and is placed into a vector containing the target DNA sequence. After expressing the library of variants in an appropriate organism, the cells are lysed and the resulting variant-plasmid complexes can be subjected to selection by exploiting a functional property of the variant proteins, such as binding affinity for a specific target. For example, Samuelson and colleagues generated a library of the *N*-glycan binding protein Fbs1 fused to NF-κB1, and used beads with fetuin (a serum glycoprotein containing complex sialylated *N*-glycans) to select Fbs1 variants with improved binding to a broad range of *N*-glycans.^90^

To make even larger libraries, is also possible to link the protein variants to their corresponding mRNA or DNA, *in vitro*. One of the first methodologies to achieve this linking was ribosome display (Fig. 7c), developed in 1997 by Hanes and Plückthun^91^ based on the previously devised polysome display.^92^ These techniques were mostly applied to the selection of proteins with enhanced binding properties, such as antibodies, and used stop codons to stall ribosomes on mRNA, and ice/magnesium to stabilise phenotype–genotype linkage. Alternatively, protein and mRNA can be directly linked with mRNA display.^93^ Modern equivalents such as CIS-display^94,95^ use *cis*-acting DNA-binding proteins (repA) to stably link each displayed protein to its coding gene. Such systems can handle some of the largest library sizes ever reported: >10^12^ variants, because no cells or transformation steps are required.

#### Growth coupling techniques

In growth coupling approaches, cell fitness is linked to the target property in the biomolecule of interest. Growth coupling techniques allow competition between variants and thus true evolution, when combined with *in vivo* mutagenesis. However, it is not always possible to couple a desired biological function to growth, and function of the evolved biomolecule can lower cell fitness. Nonetheless, proteins conferring antibiotic resistance are easily linked to increased survival rate by simply adding the antibiotic to the culture medium (Fig. 7d). Several examples exist where variants of natural antibiotic resistances have been evolved to confer enhanced or novel protection against a particular antibiotic, including proteins such as efflux pumps and β-lactamases.^96–99^ Similarly, enzymes involved in essential metabolic reactions can be selected by using auxotrophic mutants (Fig. 7e). For example, Boersma *et al.* used an aspartate-auxotrophic *E. coli* strain to select an enantioselective variant of *Bacillus subtilis* lipase A with preference towards the (S) isomer of 1,2-*O*-isopropylidene-*sn*-glycerol, a precursor for the synthesis of β-adrenoceptor antagonists.^100^

It is also possible to indirectly couple the activity of the biomolecule of interest to increased survival (Fig. 7f). For example, in the QUEST method, the selection marker is placed under a synthetic transcriptional activator comprising the AraC DNA-binding domain and a domain able to bind both a molecule inducing dimerization, and the substrate of the enzyme of interest, which compete with each other. The selection marker is only expressed upon transcription factor dimerization and conversion of the substrate to a product by the enzyme of interest.^101^ The scope of applicability of transcription factors as biosensors leading to the expression of a gene linked to survival (or even a fluorescent product for screening-based methods) is dependent on the availability of a transcription factor able to respond with high enough specificity to the presence of a desired product.^102^ While the repertoire of naturally available transcription factors is not enough to cover a wide range of target products, novel allosteric transcription factors have been engineered through directed evolution, most typically with FACS.^103^

There have also been successful attempts at mimicking the growth coupling principle by using *in vitro* compartmentalization. This is better suited to select enzymes which can promote the replication or transcription of their coding sequences. For example, Holliger and collaborators compartmentalised coding sequences for DNA polymerases in a water-in-oil emulsion and provided flanking primers and dNTPs within each aqueous compartment.^104^ They then performed PCR cycles, where a higher yield of DNA was obtained in compartments with the most active polymerases, resulting in enrichment of the sequences encoding the 'fittest' variants. By performing several rounds of selection at increasingly higher temperatures or heparin concentrations, they managed to obtain Taq polymerase variants with enhanced thermal stability and reduced heparin inhibition.

## Emerging novel paradigms in directed evolution

Recently, techniques coupling mutagenesis and selection under constant flow have been devised, enabling continuous directed evolution. Advantageously, this is >100 times faster than rounds of conventional batch selection because each cell generation can potentially provide a round of selection. One of the most famous examples is phage-assisted continuous evolution (PACE), developed by the laboratory of David Liu.^24^ In the PACE system, the evolving gene of interest restores the activity of a missing gene (gene III of the M13 bacteriophage), essential gene for phage replication. Evolving phages infect a constant flow of host cells carrying an inducible mutagenesis plasmid^26^ and an accessory plasmid. The latter responds to evolving gene variants to induce the essential gene III, so completing the phage life cycle. Thus, the system allows for the continuous evolution of the gene of interest as long as its activity can be linked to the expression of gIII. A recent variant technique (PACEmid) adapted the method to phagemids, allowing large starting-library generation.^105^ The method was illustrated by evolving the smallest dual transcription factor reported to date: a 63-amino acid peptide.^27^

Continuous directed evolution is best suited to applications where it is straightforward to link desired property to increased survival of cells. While continuous *in vivo* evolution techniques have been devised as previously discussed, the identification and isolation of the variants of interest remains a central bottleneck.^106^ This has limited its practical application, specially to small molecule biocatalysts. Indeed, only a few examples of application of PACE-like protocols to this type of cases have been described.^107,108^ Therefore, novel selection methods allowing for such linking to be established for a wider range of properties and biomolecules must be developed, to expand the scope of continuous evolution approaches. Novel selection methods recently developed based on direct or indirect regeneration of essential cofactors as a consequence of enzymatic activities might provide an useful tool in this direction.^109^

**Fig. 8:**
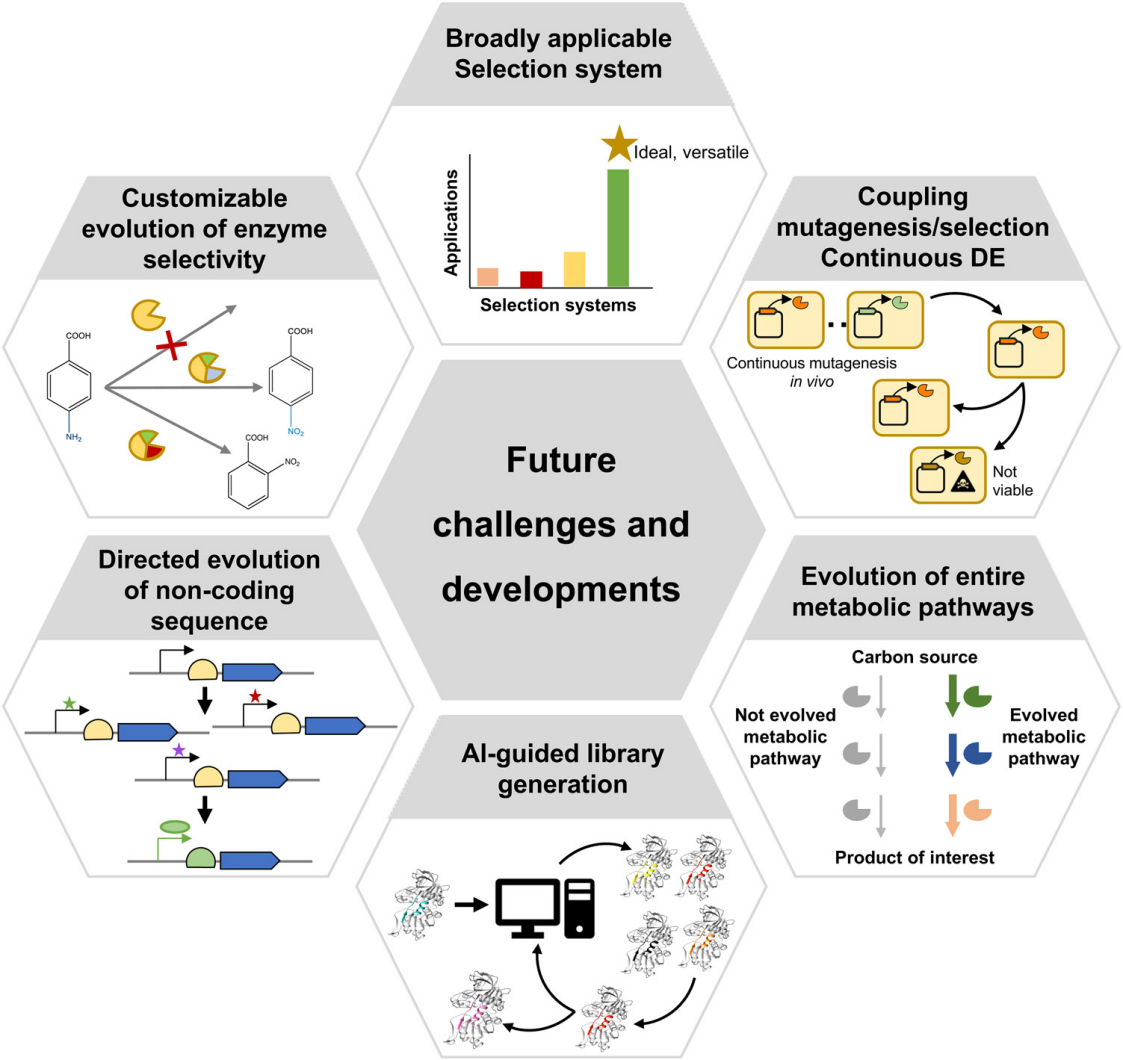
Summary of main challenges and future developments of directed evolution.

Directed evolution is moving towards the evolution of ever more complex biomolecules and systems (Fig. 8). Metalloenzymes are examples, which offer an ample range of catalysed reactions thanks to their metallic cofactors. Artificial metalloenzymes have been developed either using natural metalloenzymes as the starting point, or by engineering protein backbones to incorporate metal cofactors.^110^ In both cases, evolving novel metalloenzymes poses additional challenges, due to the requirement that the cofactor must be efficiently produced or incorporated by the expression system, and the toxicity or propensity to react with other metabolites (such as glutathione) of some metal cofactors, which can inactivate them. Such issues can be partially overcome by secreting candidate variants to the periplasmic space through the fusion of a signal peptide, as demonstrated by the evolution of a ruthenium-binding olefin “metathase”.^110^ It is also possible to perform bioconjugation of the required cofactor after expressing the apo protein and lysing cells, as demonstrated for the case of evolution of an improved cyclopropanase dependent on a dirhodium cofactor with random mutagenesis not necessarily focused on the active site.^111^ Alternatively, cells expressing transporters that import an exogenously provided cofactor provide another viable strategy, as long as cofactor toxicity is not too high. This strategy was successfully applied for the evolution of a novel cytochrome P450 variant with an iridium-containing heme-like cofactor able to produce chiral amines enantioselectively.^112^

Of increasing interest is also the evolution of entire metabolic pathways, where a set of enzymes work together towards the generation of a desired product (Fig. 8). A possible approach is to individually evolve enzymes as required for a novel, rationally-designed synthetic pathway to perform *in vitro* synthesis through a biocatalytic cascade, as shown for the manufacturing of islatravir.^113^ However, complete pathway optimization requires not only evolving the individual enzymes in the pathway, but also their arrangement and regulation. For example, Ajikumar *et al.* split a taxadiene production pathway into two modules and screened for different combinations of promoters and replication origins to find the optimal expression level and copy number of each module, thus maximizing taxadiene yield.^114^ Similar modular optimization approaches have since been applied to a variety of metabolic pathways.^115^ Simultaneously, more specialised mutagenesis methods aimed at assembling and optimising combinatorial expression libraries have been developed, such as Start-Stop Assembly^116^ or the Yeast Toolkit for Modular Assembly.^117^ However, the application of such methodologies to directed evolution has, so far, remain relatively limited. This is partly due to the fact that the effect of optimization of combinations of regulatory, non-coding elements can only be discerned *in vivo* (Fig. 8), and therefore the generated libraries must be subjected to selection. Given that, as previously mentioned, no selection methodology widely applicable to a large range of pathways is available, the extent to which such combinatorial libraries can be employed for directed evolution is constrained.

An extreme case is the application of directed evolution to whole genomes. One of the most popular methods for performing genome-scale directed evolution is Adaptive Laboratory Evolution (ALE), where microorganisms are cultured under controlled, specific conditions that enable the selection of phenotypes associated with improved growth under the chosen environment. A typical application example is the selection of *E. coli* strains with increased resistance to high temperatures by growing them at 42.2 °C during multiple generations.^118^ This has been successfully attempted up to 50 °C with libraries of transcription network rewirings as the source of variation.^119^

More recently, mutagenesis methods for genome-scale directed evolution have been devised, including several techniques based on Multiplex Automated Genome Engineering (MAGE) which employ combinations of multiple oligonucleotides to target up to thousands of genomic locations simultaneously.^120,121^ The potential of such approaches to develop new variant organisms serving as optimised whole-cell catalysts was demonstrated by Wang *et al.*, who managed to obtain an *E. coli* strain overproducing lycopene.^122^ The latest approaches employ a combination of RNA interference and CRISPR/Cas9 systems, to achieve both knockdown and activation of genes within a single cell, showing promising results in the selection of yeast strains with multiple phenotypes, such as cellulase expression and isobutanol production.^123^

## Conclusion

Over the past few decades, directed evolution has clearly demonstrated its potential to develop variants of biomolecules with novel or enhanced properties. Many cases of successful application of directed evolution have been possible thanks to the development of myriad techniques for library generation and variant identification. While efficient genetic diversification can be achieved relatively easily with modern molecular biology and chemical synthesis techniques, as well as AI-assisted computational tools, variant identification often remains as a major bottleneck. A tool that is current lacking is a selection system broadly applicable to a wider range of biomolecules, especially enzymes acting on small molecules, and even more complex systems such as cellular pathways and non-coding sequences. Such a system could be coupled to *in vivo* mutagenesis to enable real-time automated enzyme evolution. Ultimately, this would provide a solution to two of the current major challenges of directed evolution: widening applicability to multiple types of biomolecules and properties while minimising human intervention.

## Conflicts of interest

There are no conflicts to declare.

## Supplementary Material
